# Talin in mechanotransduction and mechanomemory at a glance

**DOI:** 10.1242/jcs.258749

**Published:** 2021-10-28

**Authors:** Benjamin T. Goult, Nicholas H. Brown, Martin A. Schwartz

**Affiliations:** 1 School of Biosciences, University of Kent, Canterbury, Kent CT2 7NJ, UK; 2 Department of Physiology, Development and Neuroscience, University of Cambridge, Downing St., Cambridge CB2 1DY, UK; 3 Yale Cardiovascular Research Center, Yale University School of Medicine, New Haven, CT 06511, USA

**Keywords:** Integrin, Mechanobiology, Mechanomemory, Mechanotransduction, Memory, MeshCODE, Talin, TLN1, TLN2

## Abstract

Talins are cytoskeletal linker proteins that consist of an N-terminal head domain, a flexible neck region and a C-terminal rod domain made of 13 helical bundles. The head domain binds integrin β-subunit cytoplasmic tails, which triggers integrin conformational activation to increase affinity for extracellular matrix proteins. The rod domain links to actin filaments inside the cell to transmit mechanical loads and serves as a mechanosensitive signalling hub for the recruitment of many other proteins. The α-helical bundles function as force-dependent switches – proteins that interact with folded bundles are displaced when force induces unfolding, exposing previously cryptic binding sites for other ligands. This leads to the notion of a talin code. In this Cell Science at a Glance article and the accompanying poster, we propose that the multiple switches within the talin rod function to process and store time- and force-dependent mechanical and chemical information.

## Abstract (precis)

**Summary:** Talin contains 13 force-dependent binary switches; this Cell at a Glance paper describes talin as a memory molecule where these switches confer molecular memory and information-processing functions.

## Introduction

Talin, the principal protein linking integrins and F-actin, has emerged as a key mechano-effector protein for integrin-mediated adhesion to the extracellular matrix (ECM) (Critchley, 2009; Klapholz and Brown, 2017). Talin is a large (270 kDa) multidomain cytosolic protein composed of an N-terminal FERM domain ‘head’ coupled to a large flexible rod domain consisting of 13 sequential α-helical bundles (R1–R13; see poster). The FERM domain binds directly to the cytoplasmic domains of integrin β-subunits, increasing integrin affinity for ECM ligands (Calderwood et al., 2013; Tadokoro et al., 2003). The 13 α-helical bundles (R1–R13) of the talin rod are arranged like beads on a string (Goult et al., 2013b) and connect to F-actin via both direct actin-binding sites and through vinculin. Other interactors include Rap1-interacting adapter molecule (RIAM; also known as APBB1IP), deleted in liver cancer 1 (DLC1), and cyclin dependent kinase 1 (CDK1) (see poster and Box 1 for more details). At the C-terminus of the talin rod is a dimerisation domain, which forms an antiparallel dimer with another talin molecule (Gingras et al., 2008). Talin in the cytoplasm adopts a closed, autoinhibited conformation (Dedden et al., 2019; Goksoy et al., 2008; Goult et al., 2013a), whose structure was recently solved by cryoelectron microscopy (PDB ID 6R9T; Dedden et al., 2019). A model of full-length talin in an extended conformation based on nuclear magnetic resonance (NMR) and crystallographic analyses of the subdomain structures has been proposed (Goult et al., 2013b) (see poster), which provides a map of the binding sites and a way to consider the interactions, mechanical properties and functionalities of talin.

**Box 1**. The talin interactome Talin has many binding partners, which comprise a complex talin interactome (see poster).

**The talin head domain**

The head domain binds the integrin cytoplasmic tails via its FERM domain (Anthis et al., 2009; Calderwood et al., 1999; Tadokoro et al., 2003) but also interacts with phospholipids in the plasma membrane (Anthis et al., 2009; Goult et al., 2010; Saltel et al., 2009) and with the small GTPase Rap1 (Rap1a and Rap1b in humans) (Gingras et al., 2019; Goult et al., 2010; Zhu et al., 2017). There are also multiple ligands in addition to integrins that bind its F3 domain, including RIAM (Yang et al., 2014b), layilin (Wegener et al., 2008), FAK (also known as PTK2) (Lawson et al., 2012), PIPKIγ90 (also known as PIP5K1C) (Barsukov et al., 2003) and Gα13 (GNA13) (Srinivasan et al., 2015). Together, these proteins coordinate the activation state of the integrins.

**The mechanical binary switches in the talin rod**

The 13 mechanical switches of the talin rod bind a myriad of proteins (reviewed in Goult et al., 2018). These ligands can be categorised into binding to either the folded ‘0’ or the unfolded ‘1’ state (with integrin binding on R11 possibly binding to an intermediate state) (Gingras et al., 2009).

**Folded (0) rod binders**

Many of these ligands contain LD motifs, helices with a leucine aspartate motif, that bind to the talin helix bundles via a helix addition mechanism. LD-containing proteins that bind talin in this way include RIAM (Goult et al., 2013b), KANK family proteins (Bouchet et al., 2016), CDK1 (Gough et al., 2021), DLC1, paxillin (Zacharchenko et al., 2016) and tensin (Atherton et al., 2021 preprint). LD-independent folded rod binders include F-actin (Hemmings et al., 1996), synemin (Sun et al., 2008), moesin (Beaty et al., 2014) and the talin F3 domain that mediates autoinhibition (Goksoy et al., 2008; Goult et al., 2009).

**Unfolded (1) rod binders**

Currently only vinculin has been identified as binding to talin in its unfolded state. Nine of the 13 talin rod domains contain vinculin-binding sites, which are accessible when the domains are in the unfolded state (Gingras et al., 2005; Yao et al., 2016). There are currently no known ligands for the unfolded state of domains R4, R5, R9 or R12.

**Figure:**
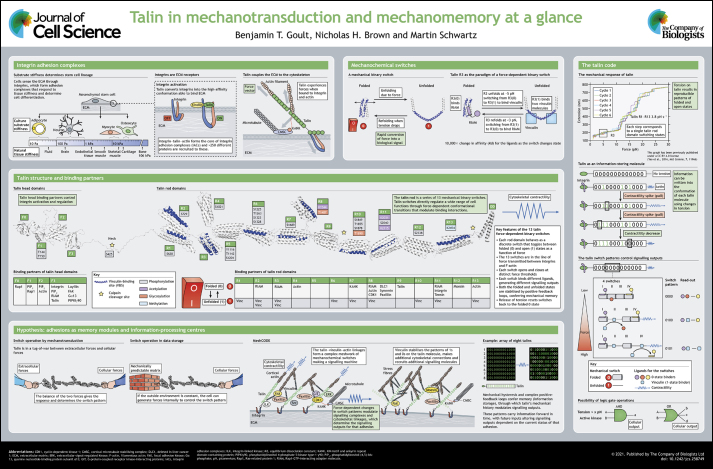

Talin arose in the amorphia lineage of eukaryotes, which includes slime moulds, fungi and animals, and is absent from other eukaryotic lineages, for example, flowering plants (Sebé-Pedrós et al., 2010). Different organisms contain between one and four talin genes, with two in humans, *TLN1* and *TLN2*. To date, all talin genes identified encode the full protein, with all domains arranged in the same order (Gough and Goult, 2018), suggesting that this arrangement is critical for function. This contrasts with other cytoskeletal linker proteins, such as filamin, which varies greatly in length (Light et al., 2012). Through gene duplication, talin has also given rise to kindlin family proteins, which contain a FERM domain but lack the rod (Ali and Khan, 2014; Meller et al., 2015) and play a key role in integrin activation (Plow and Qin, 2019; Zhu et al., 2021), as well as the actin regulatory protein talin rod domain containing protein 1 (TLNRD1) (Cowell et al., 2021). Although many helical bundles exist in nature, the talin rod fold, comprised of a five-helix bundle, appears to be unique to talins and TLNRD1.

The unique structure, force transmission and multiple conformation-dependent ligand binding activities of talin offer the potential for a remarkable array of complex, force-dependent signalling outputs. In this Cell Science at a Glance article, we briefly review talin structure, function, interactions and mechanical properties, before describing a novel view of talin as a molecular information processing and memory device that plays a key role in determining cellular responses to changes in extracellular matrix composition, organization and physical properties.

## Integrin-mediated adhesion

Integrins are the main receptors for ECM proteins, although some integrins bind transmembrane counter-receptors (reviewed in Bachmann et al., 2019; Barczyk et al., 2010; Campbell and Humphries, 2011; see poster). Integrins are heterodimers of α- and β-subunits. The short integrin β-cytoplasmic domains (‘tails’) are very similar in sequence and mediate the main cytoskeleton linkage and signalling outputs; integrin β4 is the exception, having a long cytoplasmic tail that links to intermediate filaments in hemidesmosomes (reviewed in Walko et al., 2015).

Integrins nucleate diverse adhesion classes, from small, transient nascent adhesions and focal complexes at the leading edge of cells, to larger, more stable focal adhesions that form under high mechanical loads, to podosomes and invadopodia that mediate matrix degradation and remodelling, and to fibrillar adhesions that mediate ECM assembly (Block et al., 2008; Revach et al., 2020; Zaidel-Bar et al., 2004). Within tissues*,* integrins also mediate strong, stable attachments to the ECM, such as the myotendinous junction and epidermal attachment via hemidesmosomes (Maartens and Brown, 2015; Winograd-Katz et al., 2014).

Integrins assemble intracellular protein complexes containing many cytoskeletal and signalling proteins. These complexes vary depending on the specific ligand, the integrin(s) involved, the organization, topology and mechanics of the ECM, and the expression levels of signalling and cytoskeletal proteins (Mishra and Manavathi, 2021; Seetharaman and Etienne-Manneville, 2018). The core cytoskeletal link common to nearly all integrin adhesions is provided by talin, which binds directly to the short β-subunit cytoplasmic tails (see poster). Thus, the simplest version of an integrin adhesion has just four components – the extracellular ECM ligand, the transmembrane integrin heterodimer and talin, which connects to actin filaments. Addressing the functions of the more than 250 additional components of integrin adhesions that comprise the integrin ‘adhesome’ (Chastney et al., 2020; Horton et al., 2015; Winograd-Katz et al., 2014) is an active area of research.

Binding of talin to the integrin β-tail disrupts the autoinhibitory association between the integrin α- and β-tails and promotes a conformational transition in the integrin structure that increases its affinity for extracellular ligands (Kim et al., 2011). Talin also links to actin filaments via the two actin-binding sites (ABS2 and ABS3) within the talin rod (Atherton et al., 2015) and by recruiting additional actin-binding proteins (Critchley, 2009; Goult et al., 2018; Klapholz and Brown, 2017) (see poster). We note that an additional actin-binding site ABS1 in the talin head also provides additional cytoskeletal linkages (Ciobanasu et al., 2018; Hemmings et al., 1996), but its function is not well understood. Of these, vinculin is best characterized and can bind to the 11 vinculin binding sites (VBSs) distributed throughout the talin rod (Gingras et al., 2005). In *Drosophila* at least, talin is essential for the recruitment of the remainder of the integrin-associated proteins, either directly or indirectly (Klapholz and Brown, 2017). This has led to the idea that talin forms a ‘platform’ for the assembly of an integrin adhesion complex. This design ensures that high-affinity ECM binding and connection to the cytoskeleton are functionally linked.

Once talin links integrins and actin, it transmits both cell-generated contractile forces and forces derived from externally applied strains between these components. Measured forces across talin range from a few to above 11 piconewtons (Austen et al., 2015; Driscoll et al., 2020; Kumar et al., 2016). The responses of talin to forces have four features with important consequences. First, forces stabilise the extended conformation of talin (Khan and Goult, 2019) as the head and tail are held apart by the tension, thus limiting autoinhibition mediated by head–tail interactions. Second, the binding of talin to actin and integrin shows catch-bond behaviour, that is the binding becomes stronger under moderate forces (Owen et al., 2020 preprint), which further stabilizes the activated, engaged state. Third, force unfolds the helix bundles of the talin rod domain; this simultaneously disrupts binding of proteins that bind the folded state and exposes binding sites for others (see poster and discussion below). Finally, talin rod domain unfolding exhibits hysteresis such that the force required for unfolding is higher than the force at which it refolds. For example, if a rod domain unfolds in response to a force of 10 pN it will not immediately refold when the force drops to just below 10 pN. Instead, refolding requires tension that is substantially lower (e.g. ∼1–3 pN; Yao et al., 2016). Thus, the basal physiological forces (∼5 pN) on talin within adhesions (Kumar et al., 2016) stabilise the patterns of folded and unfolded talin rod domains (Yao et al., 2016). Together, these features endow the talin molecule with mechanical memory (see poster and discussion below).

## Talin as a paradigm for a mechanotransducer

The development and maintenance of most, if not all, animal tissues and organs is guided to some extent by mechanics (Engler et al., 2006; Felsenthal and Zelzer, 2017; Urner et al., 2018). Talin and integrins play a key role in sensing and responding to mechanical forces. Cells sense both ECM stiffness and tissue strain, transmitted through the ECM, via integrins (Elosegui-Artola et al., 2018; Sun et al., 2016). In both cases, the tension on the ECM–integrin–cytoskeleton linkage is increased, leading to more integrin clustering and higher signalling outputs. Our understanding of these processes is incomplete, but the central concept is that both strain and stiffness modify the levels of tension within the integrin–cytoskeletal linkage, which alters the conformations and interactions of affected proteins. This is the crux of a molecular description of mechanotransduction, that is, how mechanical force is converted into a biochemical change, such as concentration of an effector at the adhesion site or post-translational modification of proteins.

The structure–function analysis of talin provided one of the first models for mechanotransduction. High-affinity sites for vinculin were mapped onto the talin rod but subsequent structures of the relevant domains revealed that these sites were buried (Papagrigoriou et al., 2004). This led to the hypothesis that mechanical unfolding of the talin domain was required for vinculin binding, which was subsequently confirmed by single-molecule biophysics (del Rio et al., 2009; Yao et al., 2014). This initial model was further elaborated when the Rap1 effector RIAM was found to bind to the folded R3 domain; here, force displaces RIAM and thus recruits vinculin, representing an elegant mechanical switch (Goult et al., 2013b; Lee et al., 2013; Vigouroux et al., 2020) (see poster) with a 10,000-fold change in the affinity for the two ligands as the switch changes state (Wang et al., 2019). The presence of 13 such domains within the talin rod, which unfold at different forces, is intriguing, creating opportunities for highly complex force sensing (Yao et al., 2016).

Force-independent interactions between talin and vinculin have also been reported (Atherton et al., 2020; Austen et al., 2015; Han et al., 2021; Kelley et al., 2020) although such interactions require the partial relief of autoinhibition of both proteins. This fits with the notion that these proteins must first interact in a non-mechanical manner to form the linkages for transmitting force. Once force is applied, unfolding of talin helix-bundle domains exposes vinculin-binding sites; these bind to and stabilise the active conformation of vinculin, which can also connect to F-actin and further increase the force on talin (Wang et al., 2021; Yao et al., 2014). Force also stabilizes the open states of talin and vinculin, in which the head–tail autoinhibition is disrupted. Together, these mechanisms greatly extend lifetimes of the open and engaged states for each molecule (Khan and Goult, 2019; Wang et al., 2019; Wang et al., 2021), an important form of mechanosensitivity.

## Force transmission

The mechanical behaviour of talin is critical in the complex control mechanisms that govern transmission of force between the actin cytoskeleton and the extracellular matrix. The major paradigm for force transmission between F-actin and integrins is via the ‘focal adhesion clutch’, which describes interactions between relatively stationary ligand-bound integrins and centripetally flowing F-actin near cell edges (reviewed in Elosegui-Artola et al., 2018). The bonds that transmit force in this setting are highly dynamic, with fast on and off rates. ECM stiffness alters the loading rate across these bonds, which alters internal kinetics. Importantly, stiffer substrates increase traction force and stabilize the adhesions (Elosegui-Artola et al., 2018), as does application of force by substrate stretch (Sun et al., 2016). Although these effects have been attributed to the focal adhesion clutch model for dynamic force transfer (Elosegui-Artola et al., 2018), recent work has challenged this paradigm and demonstrated a more-complex mechanism. Analysis of tension across talin together with actin dynamics identified three distinct mechanisms of force transmission, only one of which is dynamic (Driscoll et al., 2020). For newly formed cell adhesions near the cell edge, force transmission involves rapidly flowing actin driven by rapid polymerization at the edge, as described by the clutch model. However, as vinculin is recruited and actin velocity decreases, force transmission shifts to a flow-independent transfer driven by myosin contraction. This is consistent with the stabilization of vinculin-F-actin bonds under force (catch bond behaviour) (Huang et al., 2017), but not the short-lived bonds of the clutch model. Thus, vinculin contributes to the arrest of moving actin filaments and establishment of stable linkages, rather than dynamic force transmission. Importantly, the balance between these mechanisms is controlled by substrate stiffness, such that dynamic force transfer is more important on soft substrates but flow-independent force transfer dominates on stiff substrates (Driscoll et al., 2020).

A key aspect here is feedback between the forces and the sensing apparatus. Cells on stiff surfaces or subject to strain reinforce their adhesions and increase cell-generated contractile force, which in turn modifies their mechanosensing. For example, highly contractile cells require relatively stiff substrates for full spreading, whereas less contractile cells spread on softer surfaces (reviewed in Discher et al., 2005). Cells can thus adapt to environments with widely varying mechanical properties.

Talin itself appears to be one of the determinants of cellular stiffness sensing. Support for this concept comes from studies in which the stability of the third helix bundle, R3, was altered. R3 is the least-stable helix bundle, thus, the earliest to open under force (Yao et al., 2014). Indeed, R3 shows some binding to vinculin without force if vinculin is activated by other means (Kelley et al., 2020), and is completely opened under modest forces of ∼5 pN (Yao et al., 2014) (thermal forces at 37° are on the order of 1 pN; Humphrey and Delange, 2004). Introducing mutations into critical amino acids within the hydrophobic core of R3 (Goult et al., 2013b) increased the forces required for opening to ∼8 pN (Yao et al., 2014), which shifts cell spreading and force transmission toward stiffer substrates and/or high forces (Elosegui-Artola et al., 2016). Conversely, mutations that decrease the force required for R3 unfolding decrease cellular traction forces (Rahikainen et al., 2017). Thus, opening of R3 by force is a rate-limiting event in stiffness sensing and demonstrates the importance of the force-dependent switch-like behaviour of the talin rod domains in coordinating cellular processes.

## Interdependence of talin and integrins

Genetic analysis of talin and integrins supports their functional interdependence. Talin and integrins have extensive functions in development and homeostasis (Maartens and Brown, 2015; Winograd-Katz et al., 2014). Combining analysis of loss-of-function phenotypes with biochemical analysis has revealed that the majority, but not all, of integrin functions require talin. Examples of talin-independent integrin functions include the hemidesmosome integrin β4 subunit and the divergent *Drosophila* βν subunit, which do not utilize talin (Devenport and Brown, 2004). Conversely, in *Drosophila,* loss of talin in the follicular epithelium causes upregulation of cadherin, with severe developmental consequences, but loss of integrins does not, demonstrating that talin performs this function without integrin (Bécam et al., 2005). But despite these exceptions, we emphasize that the majority of integrin functions indeed require talin. In support of this, the double knockout of talin 1 and talin 2 in mouse cells results in cells that are unable to form integrin adhesions (Theodosiou et al., 2016), and in *Drosophila*, all of the adhesive functions of βPS (orthologous to β1) require talin (Brown et al., 2002). Surprisingly, the domains of talin that are needed to assist integrins to perform diverse morphogenetic processes are different, indicating that not all talin functions are required in all contexts (Klapholz et al., 2015). Thus, talin has a crucial role in mediating integrin function, but it achieves this by diverse mechanisms.

## The talin code – talin as a mechanosensitive signalling hub

The conserved structure of the talin rod with 13 linearly arranged mechanical switches that open under different levels of tension introduces opportunities for complex, time-dependent effects (see poster). Basic physical principles require that ligands that bind a domain in its open state will stabilize that conformation even after tension goes down. For example, binding of vinculin to an exposed VBS stabilizes that rod domain in its open state even after tension is drastically reduced (Yao et al., 2014). Vinculin binding also creates links to F-actin, which supports higher force transmission (Kumar et al., 2016). Higher tension then further increases the forces on adjacent domains. Thus, there are multiple molecular mechanisms that, once talin is opened and under tension, would tend to maintain talin domains in an open, high-tension state. Conversely, ligands that bind folded talin helix bundles will stabilize that conformation and increase the force required for opening, subject to the expression and affinity of ligands. Closed states are thus also subject to positive feedback. These mechanisms that stabilize open or closed states represent a form of molecular memory.

State-dependent ligand binding also offers opportunities for signal transduction. For example, recruitment of a kinase or GTPase to talin would initiate signalling, or sequester a protein away from its site of action to terminate signalling. Indeed, many such regulators bind talin (Goult et al., 2018) (see poster and Box 1). The talin rod domain structure therefore offers potential for signalling outputs with complex time dependence, where past tension events determine future signalling outputs.

Post-translational modifications, such as phosphorylation of exposed residues in unfolded bundles, might mediate longer-term stabilization of the open state (see poster). For instance, the talin switch domains often contain serine and threonine residues that are buried in the hydrophobic core. Upon exposure by domain unfolding, these sites become susceptible to modifications such as phosphorylation, which then limit refolding. Talin is also modified by proteolytic cleavage by calpain proteases (Bate et al., 2012; Franco et al., 2004), which is mechanosensitive (Saxena et al., 2017), providing another regulatory axis for mechanomemory.

Finally, we note that signalling reactions may depend on the folded or unfolded states of spatially proximate domains. Bringing together an enzyme and substrate on adjacent domains provides an example. This type of interaction introduces the potential for ‘AND/OR’ logic gates. Together, the talin code might integrate a complex mechanosensory axis with the classical signalling pathways of cells.

## Can mechanical linkages store and process information?

All information processing requires, first, a means of establishing stable, switchable states, or, in other words, memory (Gallistel and King, 2009). Cellular mechanical memory has been reported. Culturing several types of tissue stem cells on soft or stiff substrates has been shown to give rise to patterns of gene expression that persist for weeks after switching to the opposite substrate (Dunham et al., 2020; Li et al., 2017; Yang et al., 2014a). In one case, activation on stiff substrates was essentially irreversible (Yang et al., 2014a). We hypothesize that talin may play a role in these processes. The positive-feedback loops described in the previous section offer means for establishing long-lived states among the talin rod switches, which is, in essence, mechanical memory. Although it remains to be demonstrated that patterns of open and closed talin switches can persist for weeks or months, extending the duration of talin-dependent signals to longer time frames may be sufficient to induce epigenetic imprinting or other long-lived mechanisms of regulation.

Importantly, experimental data support the notion that talin is more stable than is commonly recognized. Fluorescent recovery after photobleaching (FRAP) showed that ∼60% of the talin is immobile during FRAP time frames of several minutes (Stutchbury et al., 2017). Within tissues, talin has been shown to turn over at very low rates, remaining stable for many hours (Hákonardóttir et al., 2015; Lemke et al., 2019).

The talin code model proposes that the 13 talin helix-bundle domains function as binary switches that can transition between a folded ‘0 state’ and an unfolded ‘1 state’ (see poster). If these conformational states determine signalling outputs, the switch patterns can be considered to encode information in a binary format. For example, tension above a threshold force (x pN) together with an active kinase (AND) gives rise to output a, whereas either tension or kinase activation alone (OR) result in different outputs (e.g. b,c) (see poster). For example, phosphorylation of talin can alter the mechanical stability of the phosphorylated domain (Gough et al., 2021), altering the order in which the domains unfold and, by extension, the molecules recruited under equivalent tension conditions.

More-complex relationships seem plausible. Binding partners on nearby rod domains might interact via enzyme–substrate or other mechanisms. In that case, the conformational states of different domains will influence each other's outputs. For example, if domains X and Y are both closed and both bound to their respective ligands A and B, then A phosphorylates B, which activates downstream pathway C. This sequence can be modulated by mechanical history, by post-translational modifications of X and Y, by expression levels of A and B, and by expression or affinity of other ligands that stabilize the bound or open states of X and Y. The possibilities thus extend to beyond simple logic gates (AND versus OR) to more-complex logic with multiple dependencies and multiple outputs.

## Conclusions and perspectives

In this article, we have established the talin code model based on three properties – memory, signalling and information processing. These features lead to the concept that cell–ECM adhesions can both store and process information.

The simplest version of the hypothesis that talin encodes information is that adhesions in mechanically stable environments store information in binary switches provided by individual talin helix bundle domains. Each adhesion would serve as a memory module and information-processing centre. At the core of each adhesive structure will be a scaffold comprised of talin molecules, with its composition and signalling outputs dictated by the binary switch patterns of each talin molecule in that macromolecular complex. The talin molecules form the core of a meshwork of mechanosensitive molecules, intricately linking integrins to the force generation machinery of the cells. This meshwork of mechanical switches at an adhesion has the capacity to serve as a mechanical code, termed a MeshCODE (Goult, 2021). The binary patterns stored in each talin molecule that are generated by forces acting on the talin molecule are highly reproducible (Yao et al., 2016) and can be altered by phosphorylation (Gough et al., 2021). Vinculin binding to a talin VBS stabilises that helix bundle in the open conformation (Yao et al., 2014), with the lifetime of the complex tuned by the interaction of vinculin with actin and the resultant force (Chen et al., 2006; Dumbauld et al., 2013; Wang et al., 2021). In this way, vinculin stabilises individual talin rod domain helix bundles in their ‘1’ state, helping maintain these patterns and allowing robust and reproducible adhesion complexes, mechanical linkages and signalling responses. Conversely, ligands that bind the folded, ‘0’ state stabilize their target helix bundles in this conformation and promote an opposite set of signalling responses.

In this way, each talin molecule would store information as patterns of 1s and 0s, representing a type of binary coding (see poster) that governs cell behaviour. Changes in tension would switch specific domains to a new state, recruiting and/or displacing molecules from the adhesion site to alter signalling outputs (see poster). For instance, an extracellular ligand binding a cell surface receptor to activate the cells force generation machinery would increase force to update the switch patterns and the resulting signalling output. Such signals also have the potential to regulate epigenetic imprinting and other long-lived mechanisms of cellular regulation.

Having laid out these possibilities, we emphasize that these notions are highly speculative. Nevertheless, the individual elements are grounded in experimental results. The concept of individual talin molecules acting as memory molecules has been shown experimentally (Yao et al., 2016), and the ability of talin switches to recruit different signalling molecules as a function of force to control cellular processes is also known (Elosegui-Artola et al., 2016; Goult et al., 2018; Haining et al., 2018; Lee et al., 2013). However, it remains to be investigated whether cells assemble these mechanisms in a coherent way with the characteristics of information processing systems. This would point to a level of order of these cytoskeletal systems that is beyond current appreciation.

However, if the history of cell biology has taught us anything, it is that cells seldom miss an opportunity to harness the laws of physics and chemistry to their own ends. We look forward to seeing the results from critical experiments designed to test the hypotheses proposed here.

## Supplementary Material

10.1242/jcs.258749_sup1 Poster

10.1242/jcs.258749_sup2 Poster Panel 1. Integrin adhesion complexes (I)

10.1242/jcs.258749_sup3 Poster Panel 2. Integrin adhesion complexes (II)

10.1242/jcs.258749_sup4 Poster Panel 3. Mechanochemical switches

10.1242/jcs.258749_sup5 Poster Panel 4. Talin structure and binding partners (I)

10.1242/jcs.258749_sup6 Poster Panel 5. Talin structure and binding partners (I)

10.1242/jcs.258749_sup7 Poster Panel 6. The talin code (I)

10.1242/jcs.258749_sup8 Poster Panel 7. The talin code (II)

10.1242/jcs.258749_sup9 Poster Panel 8. Hypothesis: adhesions as memory modules and information-processing centres (I)

10.1242/jcs.258749_sup10 Poster Panel 9. Hypothesis: adhesions as memory modules and information-processing centres (II)

## Cell science at a glance

Individual poster panels are available for downloading at https://journals.biologists.com/jcs/article-lookup/doi/10.1242/jcs.258749#supplementary-data
