# Supplementary figures and images for: Talin in mechanotransduction and mechanomemory at a glance
Source: J Cell Sci. 2021 Oct 28;134(20):jcs258749. doi: 10.1242/jcs.258749 (PMC8697387; doi:10.1242/jcs.258749)

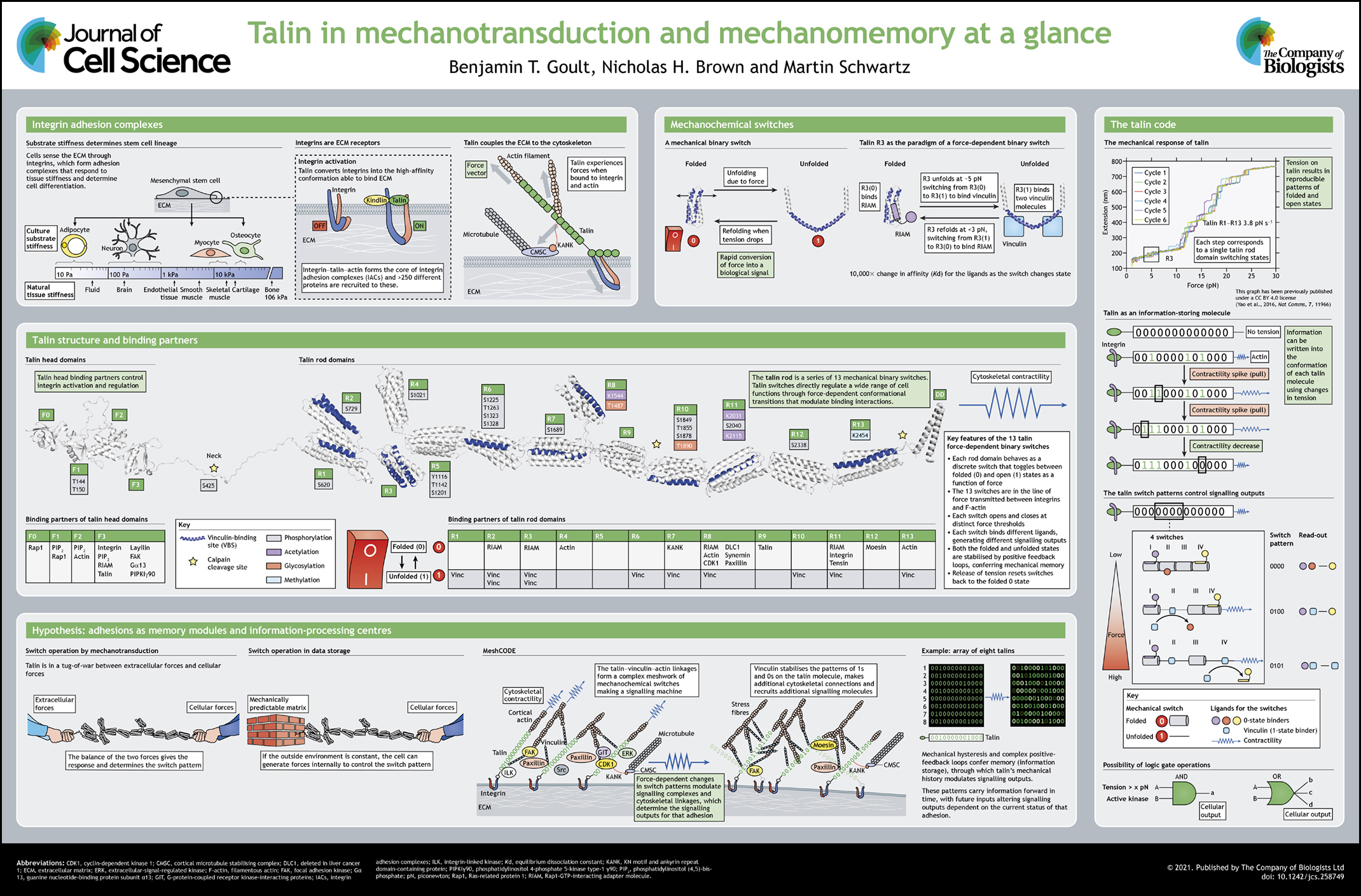

Supplement: Poster [file JCS258749supp.jpg]

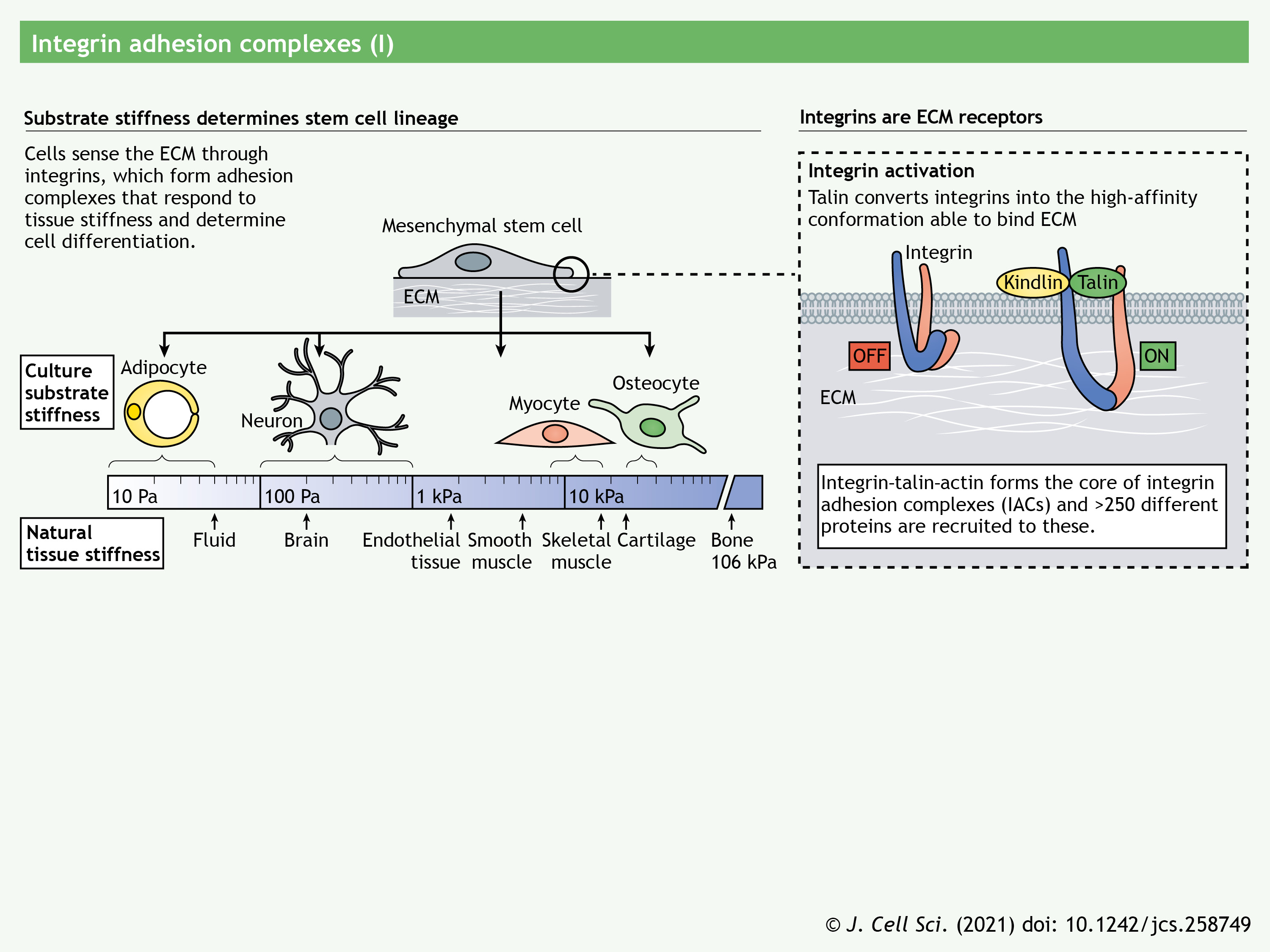

Supplement: Poster Panel 1. Integrin adhesion complexes (I) [file JCS258749supp1.jpg]

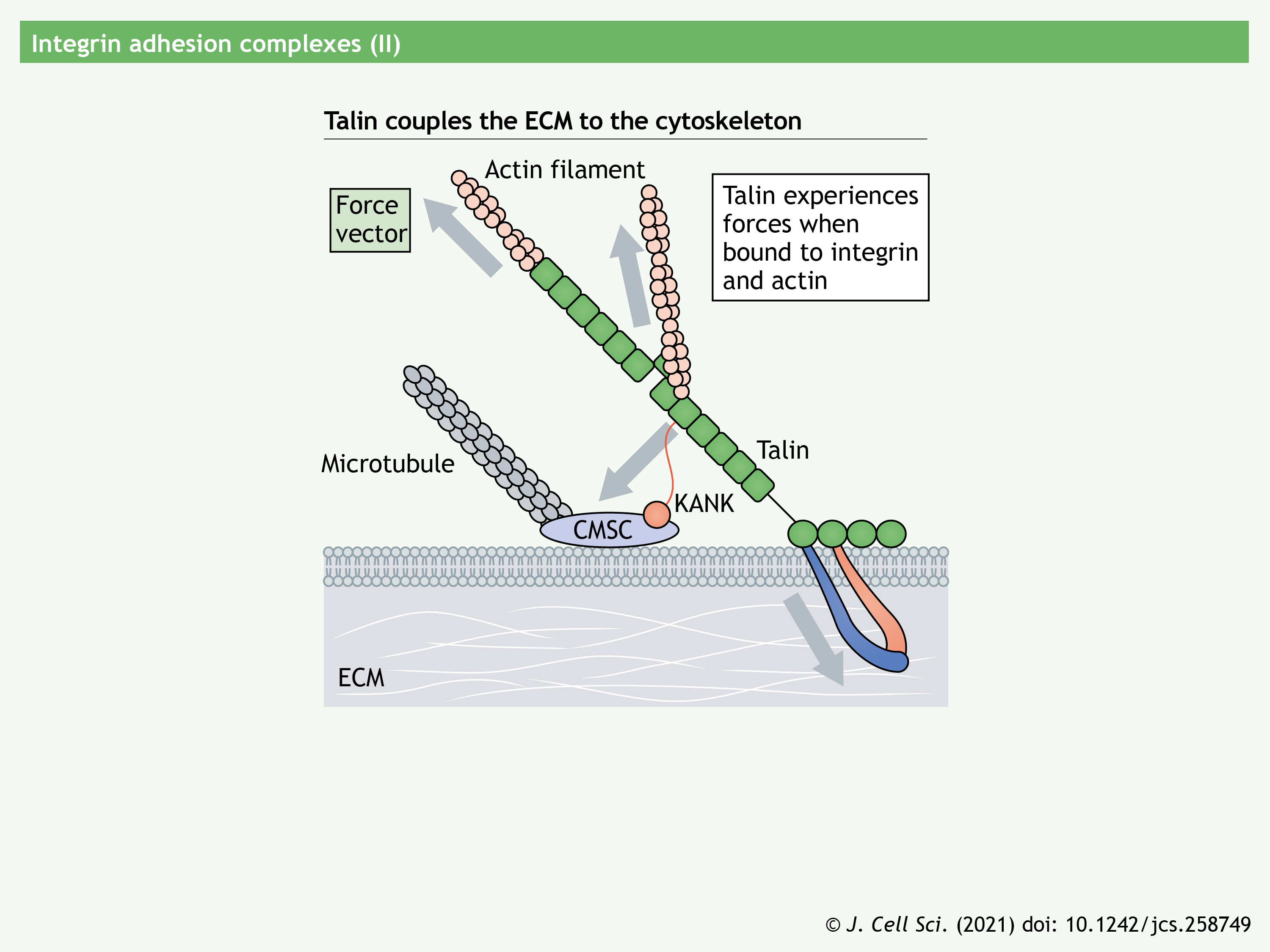

Supplement: Poster Panel 2. Integrin adhesion complexes (II) [file JCS258749supp2.jpg]

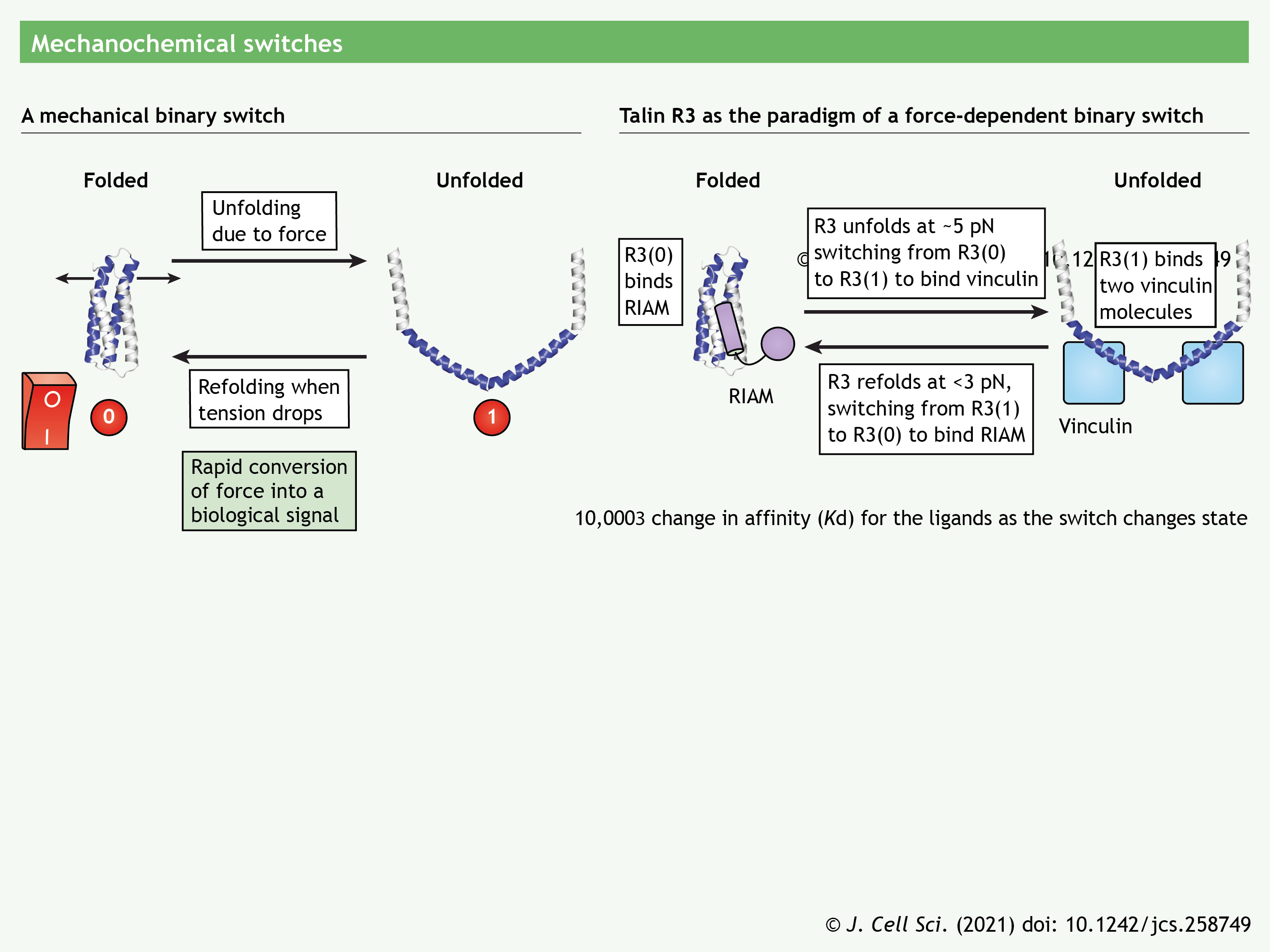

Supplement: Poster Panel 3. Mechanochemical switches [file JCS258749supp3.jpg]

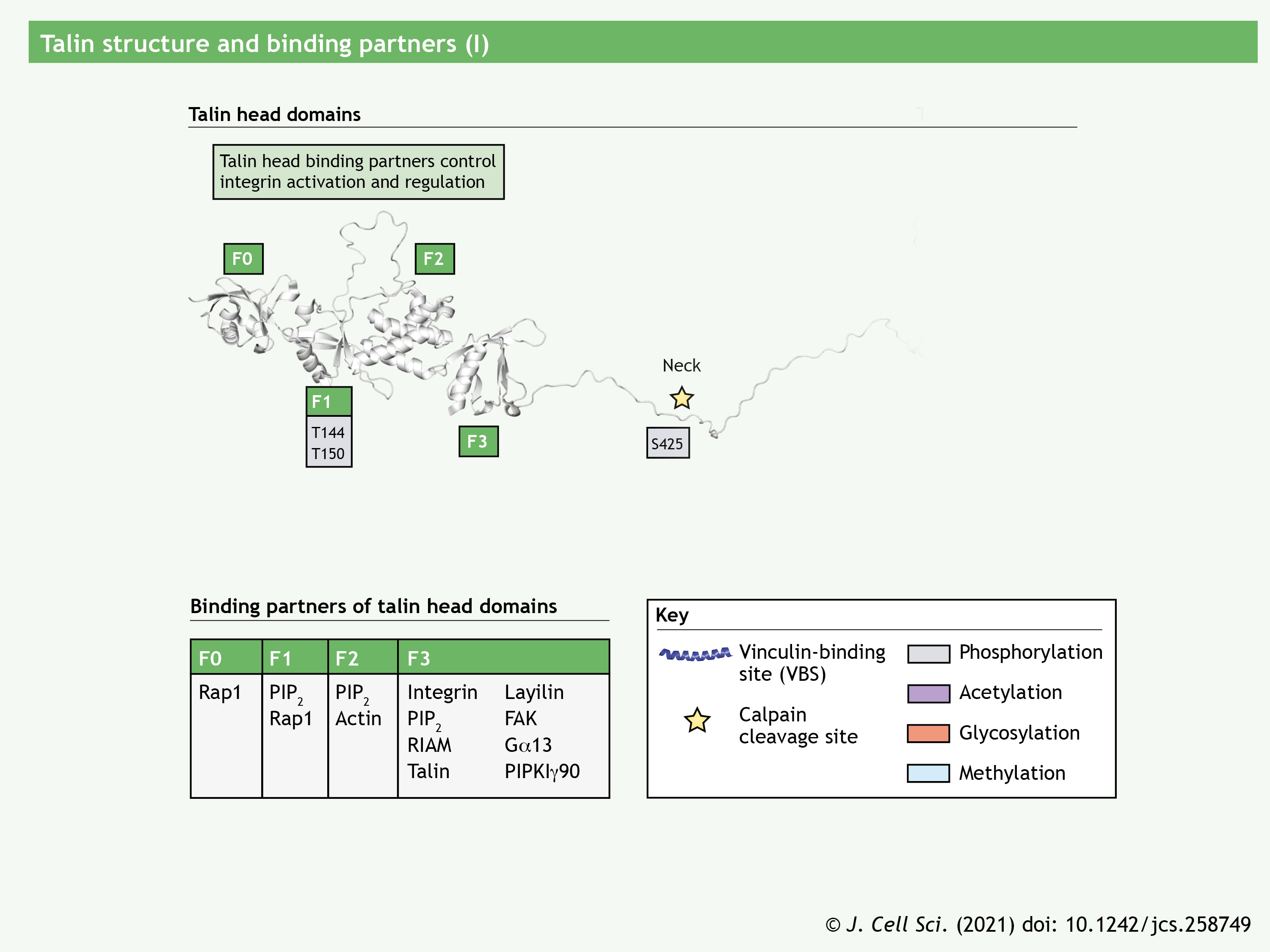

Supplement: Poster Panel 4. Talin structure and binding partners (I) [file JCS258749supp4.jpg]

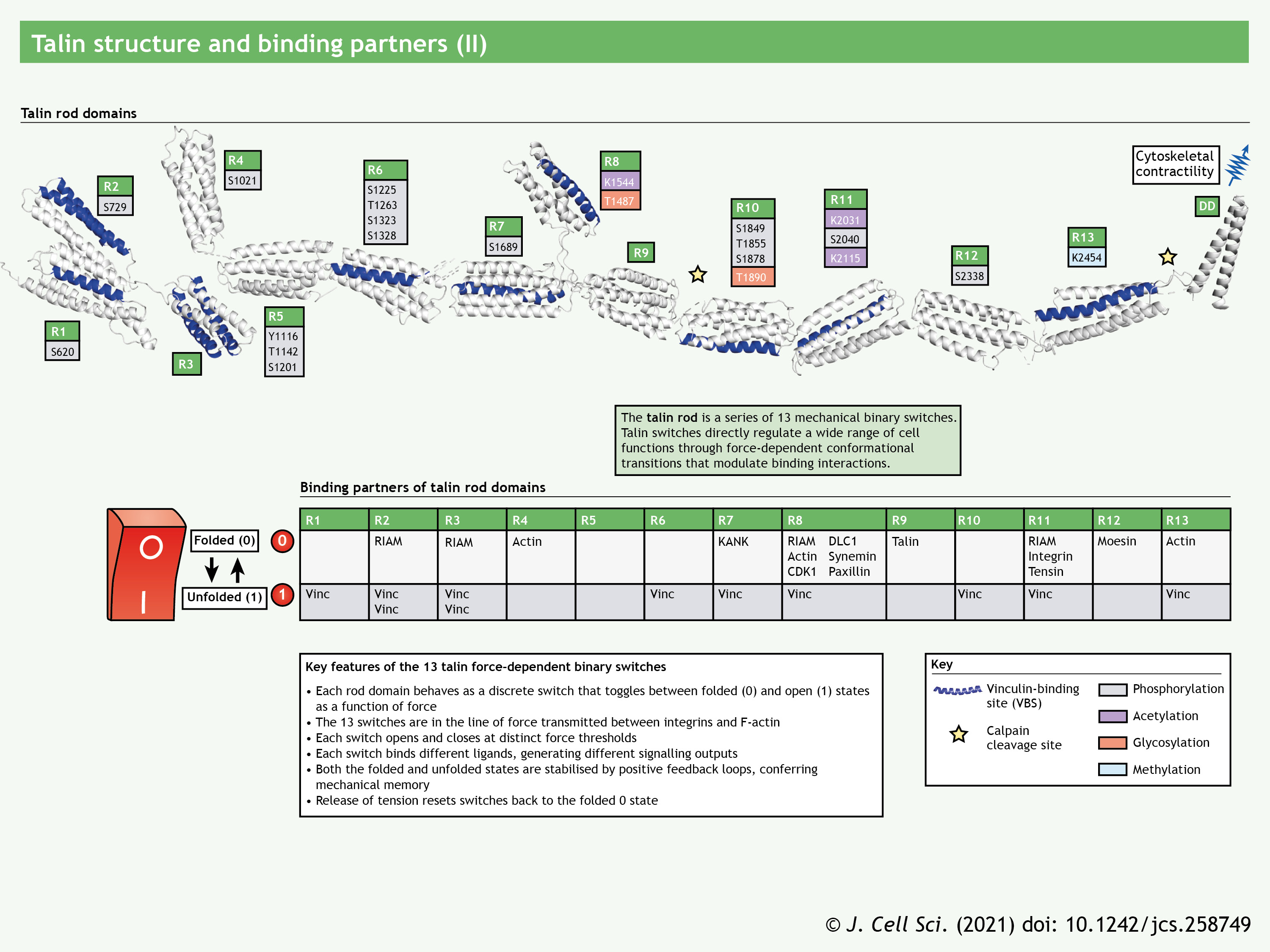

Supplement: Poster Panel 5. Talin structure and binding partners (I) [file JCS258749supp5.jpg]

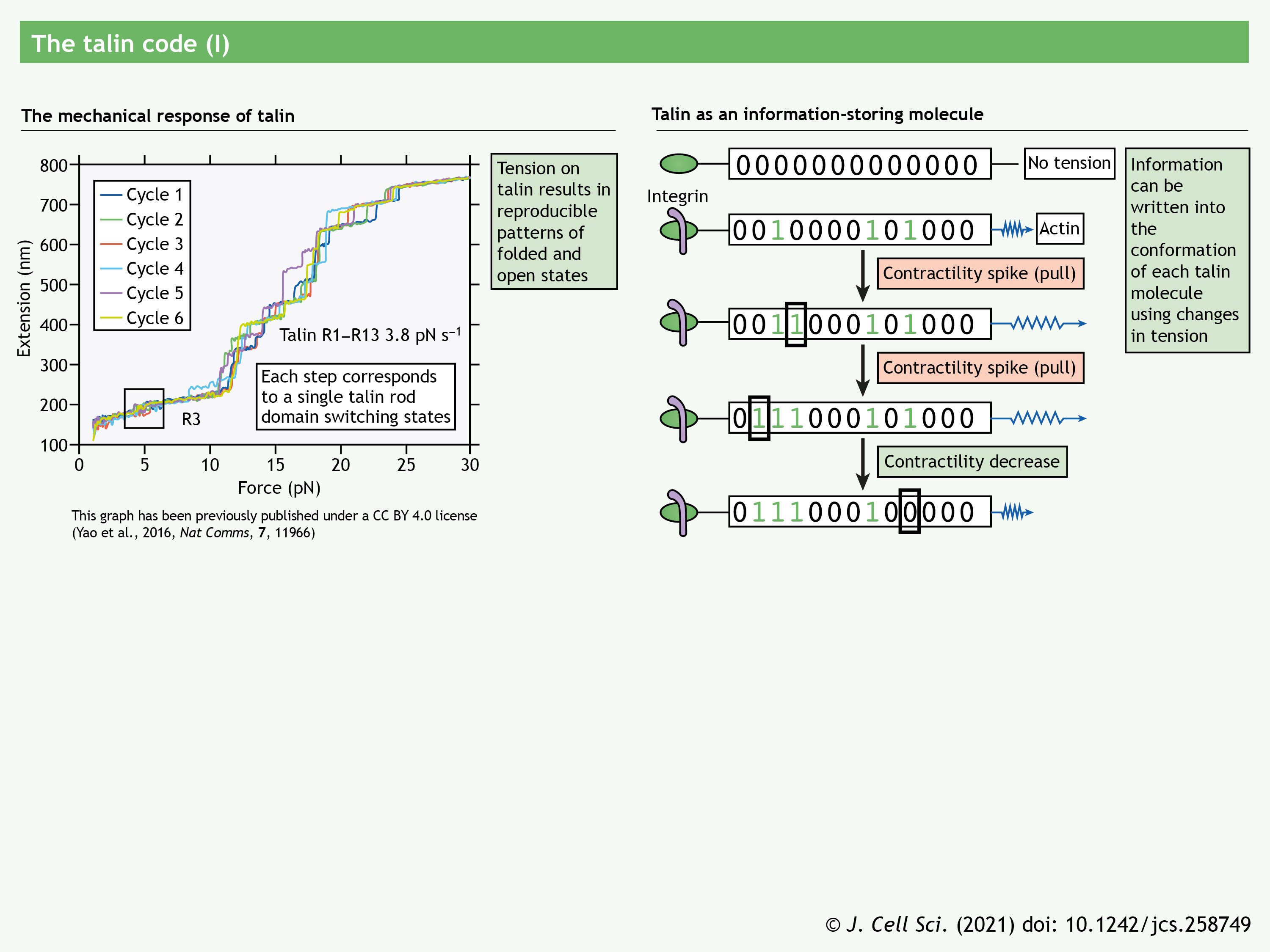

Supplement: Poster Panel 6. The talin code (I) [file JCS258749supp6.jpg]

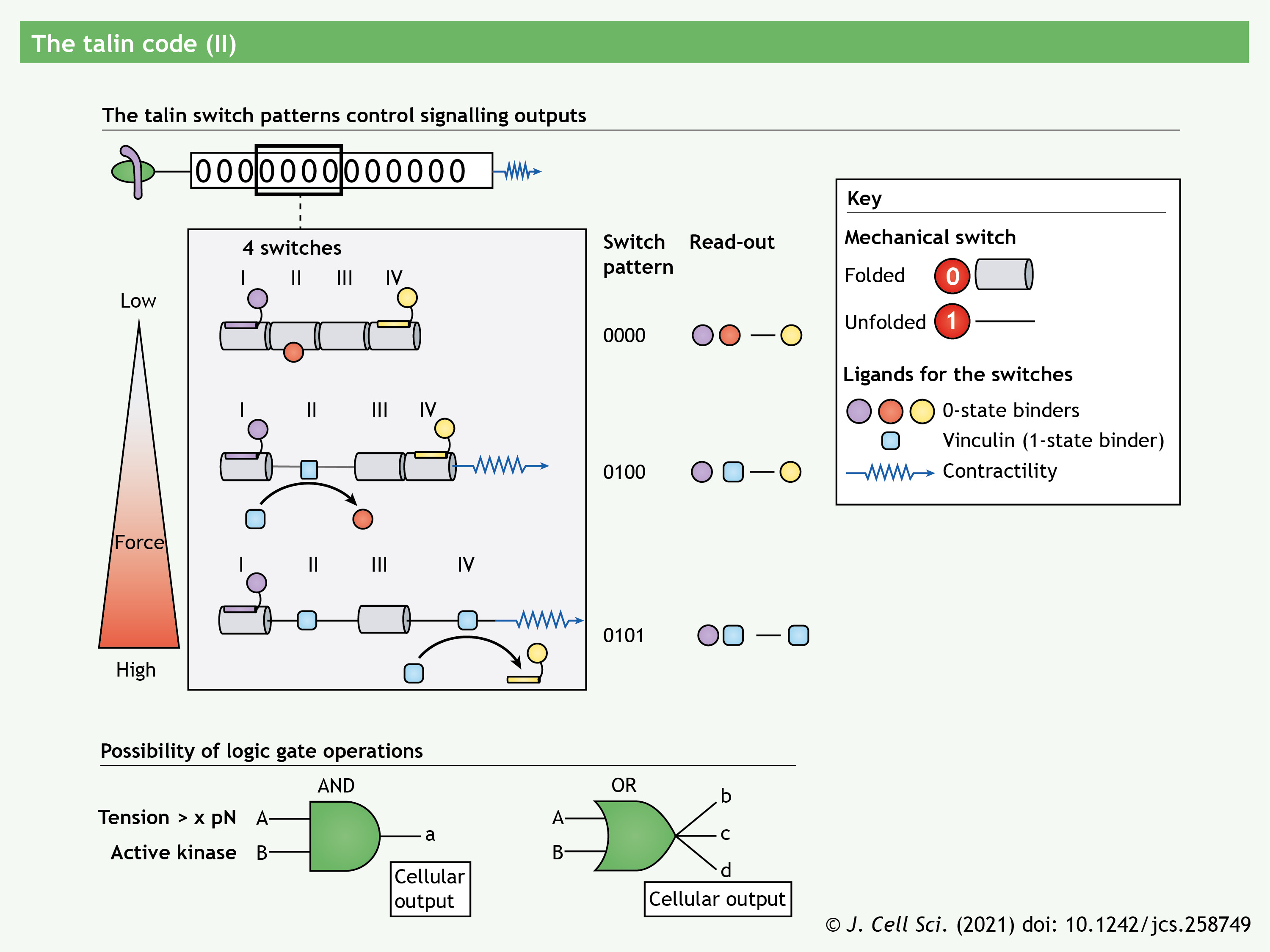

Supplement: Poster Panel 7. The talin code (II) [file JCS258749supp7.jpg]

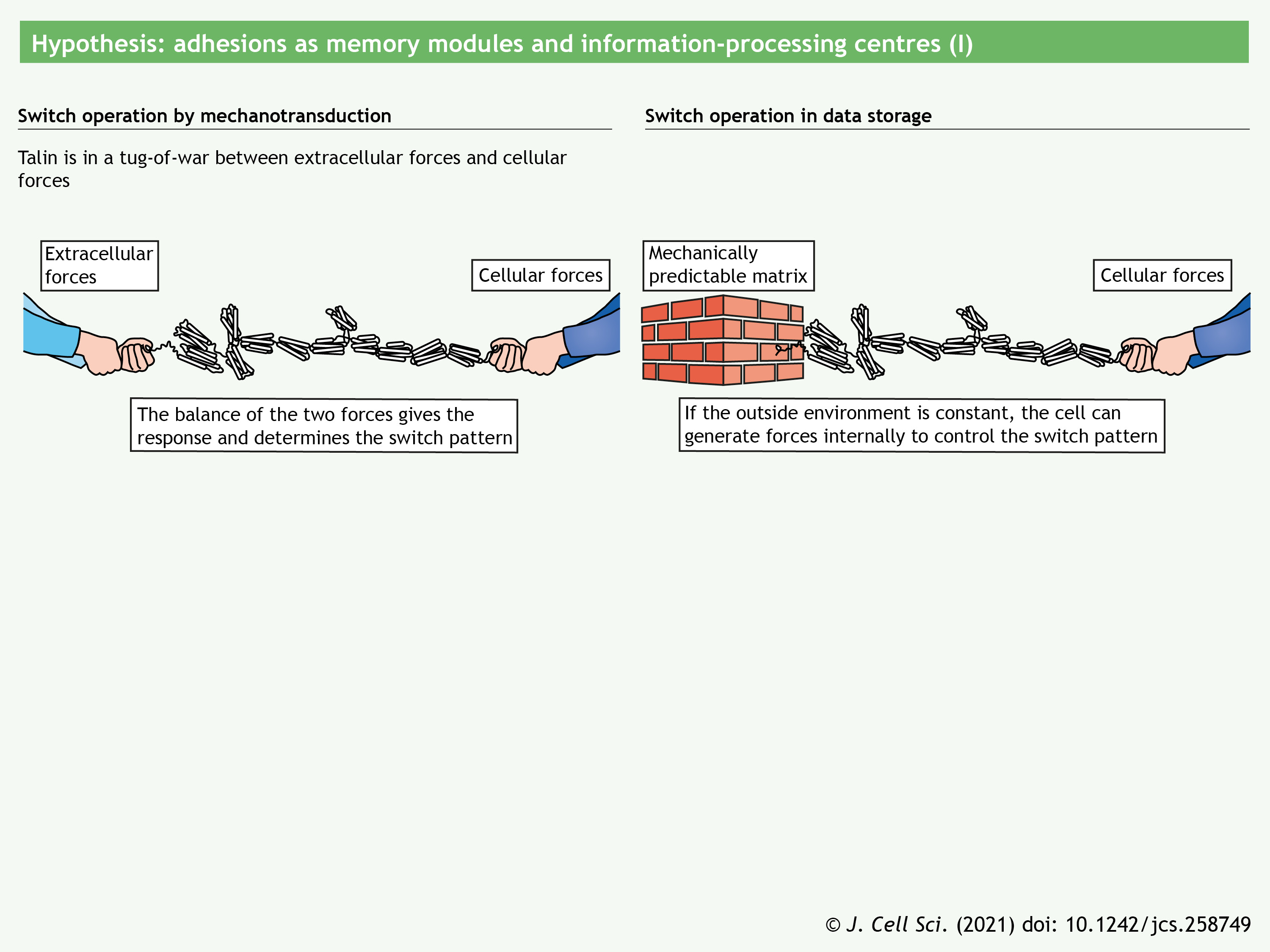

Supplement: Poster Panel 8. Hypothesis: adhesions as memory modules and information-processing centres (I) [file JCS258749supp8.jpg]

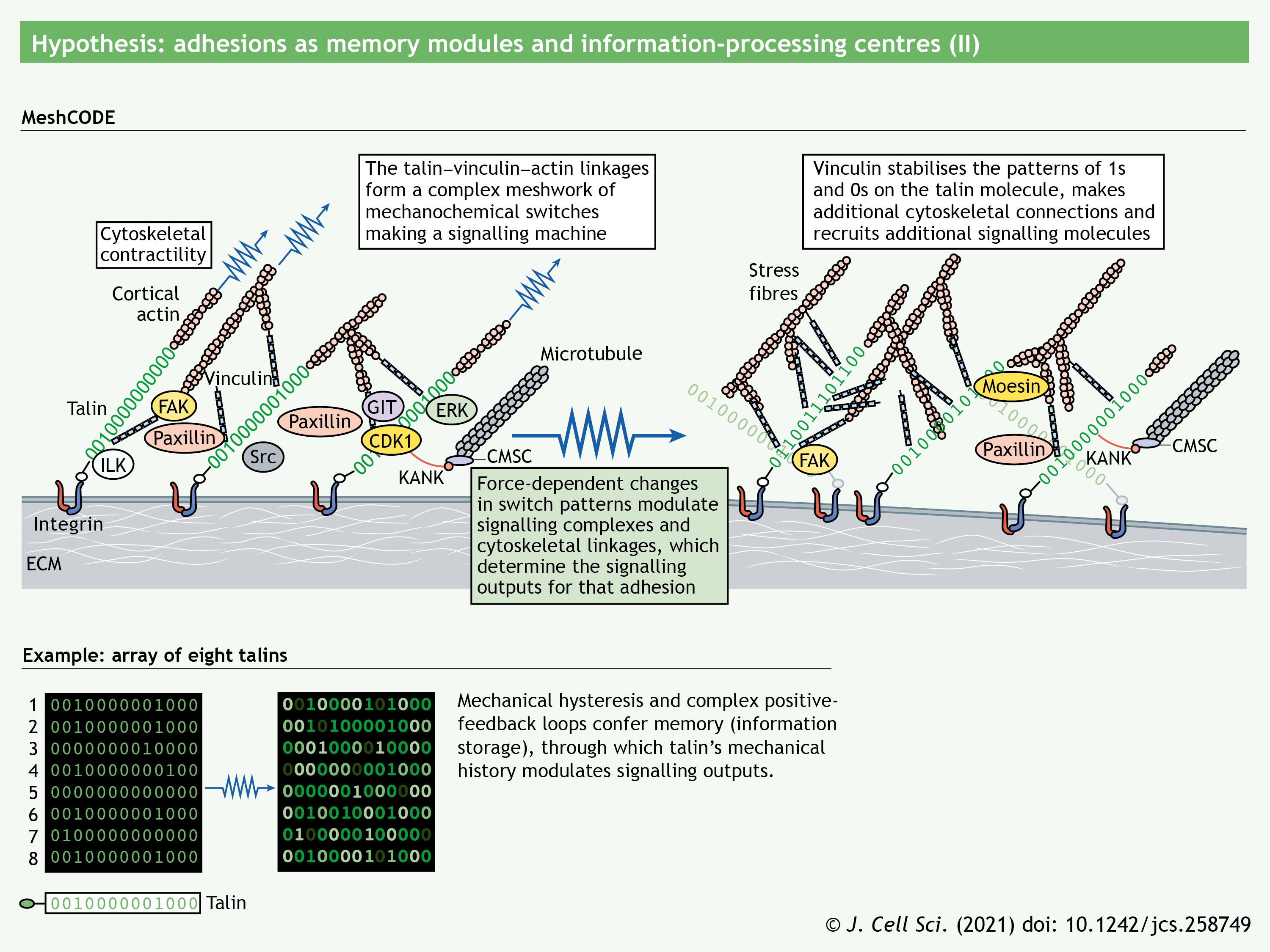

Supplement: Poster Panel 9. Hypothesis: adhesions as memory modules and information-processing centres (II) [file JCS258749supp9.jpg]
